# Levels of Abnormal Prion Protein in Deer and Elk with Chronic Wasting Disease

**DOI:** 10.3201/eid1306.070186

**Published:** 2007-06

**Authors:** Brent L. Race, Kimberly D. Meade-White, Anne Ward, Jean Jewell, Michael W. Miller, Elizabeth S. Williams, Bruce Chesebro, Richard E. Race

**Affiliations:** * Rocky Mountain Laboratories, Hamilton, Montana, USA; † University of Wyoming, Laramie, Wyoming, USA; ‡ Colorado Division of Wildlife, Fort Collins, Colorado, USA; 1 Deceased

**Keywords:** chronic wasting disease, CWD, prion, cervids, deer, elk, pathogenesis, lymphoid tissue, surveillance, immunoblot analysis, research

## Abstract

Chronic wasting disease (CWD) of deer and elk is a widespread health concern because its potential for cross-species transmission is undetermined. CWD prevalence in wild elk is much lower than its prevalence in wild deer, and whether CWD-infected deer and elk differ in ability to infect other species is unknown. Because lymphoid tissues are important in the pathogenesis of some transmissible spongiform encephalopathies such as sheep scrapie, we investigated whether CWD-affected elk and deer differ in distribution or quantity of disease-associated prion protein (PrPres) in lymphoid tissues. Immunoblot quantification of PrPres from tonsil and retropharyngeal lymph nodes showed much higher levels of PrPres in deer than in elk. This difference correlated with the natural prevalence of CWD in these species and suggested that CWD-infected deer may be more likely than elk to transmit the disease to other cervids and have a greater potential to transmit CWD to noncervids.

## Abstract 2

Infected deer may pose a higher risk than elk for disease transmission.

Chronic wasting disease (CWD) is an emerging infectious disease first recognized in the 1960s. It is a member of the transmissible spongiform encephalopathy (TSE) disease group that includes sheep scrapie, bovine spongiform encephalopathy (BSE), transmissible mink encephalopathy, and several human diseases, including kuru, Creutzfeldt-Jakob disease (CJD), and variant CJD (vCJD). Several heritable but extremely rare forms of TSE are found, most notably, Gerstmann-Straussler-Schienker syndrome (GSS) and fatal familial insomnia. CWD, like other TSE diseases, is characterized by the accumulation in neural tissues of an abnormal disease-associated prion protein designated PrPres (*1*), PrP^Sc^ (*2*), or PrP^d^ (*3*). Most TSE researchers believe that PrPres is critical in disease pathogenesis, and some evidence suggests that PrPres may itself be the infectious agent (*4*).

As recently as 1997, CWD in the wild appeared to be confined to a few counties of northeast Colorado and southeast Wyoming**.** Since then, new cases have been reported in wild deer from many additional states. The disease has recently emerged in captive elk and deer facilities scattered across the United States and Canada. Whether these new foci of infection resulted from contact with captive cervids or represent established foci of infection discovered by recent surveys is unknown. In disease-endemic areas, the prevalence of CWD in deer is variable but is often >5%. In contrast, the prevalence in wild elk is typically <1% (*5*,*6*). What factors account for different CWD prevalence in the wild between deer and elk are not known.

In some species, the infectious agent and PrPres accumulate in both lymphoid tissues and brain. The extent of lymphoid tissue involvement varies depending on the host and agent involved. In scrapie-infected sheep, lymph nodes and spleen are infected early and are directly involved in the kinetics of disease (*7*–*9*). Lymphoid tissues are likewise important in vCJD (*10*) in humans and also in some TSE mouse models (*11*–*13*). In other TSE diseases such as BSE and sporadic CJD, lymphoid tissues appear to play little or no essential role in disease pathogenesis (*14*,*15*). Knowing the extent of lymphoid tissue involvement in deer and elk might provide clues regarding modes of natural transmission in these species or the potential for transmission to other species.

PrPres in lymphoid tissues of deer (*16*) and elk (*17*) has been primarily detected by using immunohistochemical (IHC) techniques. However, with these techniques, quantification and glycoform analysis of PrPres are not possible. We were interested in determining whether PrPres found in lymphoid tissues of deer differs from PrPres found in lymphoid tissues of elk in quantity, distribution, or structural features. Immunoblot techniques enabled us to study these questions.

Surveys of CWD-infected deer and elk based on IHC or ELISA analysis of brain or retropharyngeal lymph nodes (RPLNs) have not shown differences between the 2 species that explain why CWD prevalence differs in natural settings (*18*,*19*). In our study, we sought to identify potential differences in biochemical characteristics of PrPres to explain the prevalence differences between the 2 species**.** We found that lymphoid tissues of CWD-infected deer had much greater quantities of PrPres than were detected in similar samples from elk. Furthermore, we found a wider distribution and higher incidence of positive lymphoid tissues in deer. These differences might account for the disparity in the reported prevalence of CWD in the wild between deer and elk. Our results also support previous observations that suggested CWD surveillance programs based on IHC detection of PrPres in lymphoid tissues alone may not be appropriate for elk (*5*,*18*).

## Materials and Methods

### Tissues

Brain, tonsil, spleen, and selected lymph nodes, including RPLNs, prescapular, submandibular, superficial cervical, mesenteric, popliteal, and ileocecal-colic lymph nodes, were obtained from 10 CWD-infected elk and 15 CWD-infected deer (12 mule deer and 3 white-tailed deer). Elk were derived from game farms or research facilities where they became infected by contact with CWD-infected elk, a contaminated environment, or oral inoculation. All of the elk used in this study had definite clinical cases when they were euthanized. The deer used for PrPres quantification all had confirmed clinical cases and were from research facilities where they became infected by contact with infected animals. Three of the mule deer included in Table 1 were harvested by Colorado Division of Wildlife or Wyoming Department of Game and Fish personnel. Tissues from wild uninfected deer and elk were obtained from Montana Department of Fish, Wildlife and Parks. More than 4,000 wild deer and elk from Montana have been tested for CWD with no positives found.

**Table 1:** PrPres detection by immunoblot in brain and lymphoid tissues of elk and deer*

Species	Brain	Spleen	Tonsil	Lymph node
Elk	10/10	2/10	4/9	5/10
Mule deer	12/12	2/10	9/10	12/12
White-tailed deer	3/3	1/3	2/3	3/3

*PrPres, disease-associated prion protein. Values indicate the number of animals with PrPres detected (numerator) over the number analyzed (denominator). If any PrPres was detected in a given tissue, it was considered positive for the purpose of this table. If even a single lymph node from a given animal was positive, the animal was scored as positive for that tissue. This table does not consider quantitative variation in the amount of PrPres in tissues of elk compared to deer. The denominators vary because not every tissue was available from every animal. Brain, tonsil, and lymph nodes of elk did not differ significantly from mule deer (Fisher exact test).

### PrPres Purification

Twenty-percent tissue homogenates of brain, tonsil, lymph nodes, or spleen from CWD-infected and uninfected deer and elk were made in 0.01 mmol/L Tris-HCl, pH 7.4, 0.005 mmol/L MgCl_2_ by using either disposable Konex microcentrifuge tubes (Kimble/Kontes, Vineland, NJ, USA) and matched pestles (brain) or an omni tissue homogenizer (tonsil, spleen, and lymph nodes); 75%–90% of the total tissue mass of respective lymph nodes or tonsil was homogenized. Two-hundred–milligram aliquots of the total homogenate were processed further to concentrate PrPres by using ultracentifugation and proteinase K digestion as described (*20*).

### Immunoblotting

Protein gel electrophoresis and immunoblotting were done as previously described (*21*,*22*) by using polyclonal antibody R35 (*23*) or monoclonal antibody L-42 (R-Biopharm AG, Darmstadt, Germany). L-42 reacts with PrPres from several species, including deer and elk, and has been well characterized (*24*). Blots were developed by using either an enhanced chemiluminescence (ECL) or enhanced chemifluorescence (ECF) system, according to the manufacturer’s instructions (Amersham-Pharmacia, Piscataway, NJ, USA). ECL blots were exposed to film to visualize proteins. ECF blots were scanned by using a STORM fluorescent detection system (Amersham-Pharmacia) as described previously (*25*).

### PNGaseF Digestion

Reagents and enzymes for PNGaseF treatment were purchased from New England BioLabs (Beverly, MA, USA). Reaction conditions were as recommended by the manufacturer except that denaturing of 1- to 30-mg tissue equivalents was done in a total volume of 20 μL sodium dodecyl sulfate–polyacrylamide gel electrophoresis sample buffer. Each sample was digested by using 2,500 U PNGaseF and incubated overnight at 37°C. Samples were frozen at −20°C until they were analyzed by immunoblotting.

## Results

### Quantification of PrPres in Brain, Tonsil, and RPLNs

**Figure 1:**
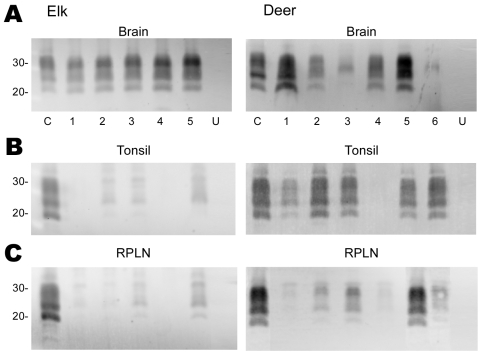
Immunoblot analysis of PrPres from chronic wasting disease (CWD)–affected elk and deer brain, tonsil, and retropharyngeal lymph node (RPLN). Panel A shows the PrPres signal from 2-mg equivalents of elk brain or 20-mg equivalents of deer brain. Individual animals are identified as 1–5 (elk) or 1–6 (deer). C denotes the reference control to which all other samples are compared and consists of 20-mg equivalents of retropharyngeal lymph node (RPLN) from a CWD–affected mule deer. Aliquots of this same control are included on all blots shown in panels B and C. Lanes labeled U in panels A, B, and C contain 20-mg equivalents of the respective tissue from uninfected elk or deer. No PrPres bands were detected when tissues from uninfected deer or elk were analyzed. In panels B and C, 20-mg equivalents of tonsil or RPLN were used. PrPres was obtained as described in Materials and Methods and the blots developed by using antibody L42 at a 0.04 μg/mL dilution and standard enhanced chemifluorescence processing. Approximate molecular weights in kd are indicated on the left side of the panels.

**Figure 2:**
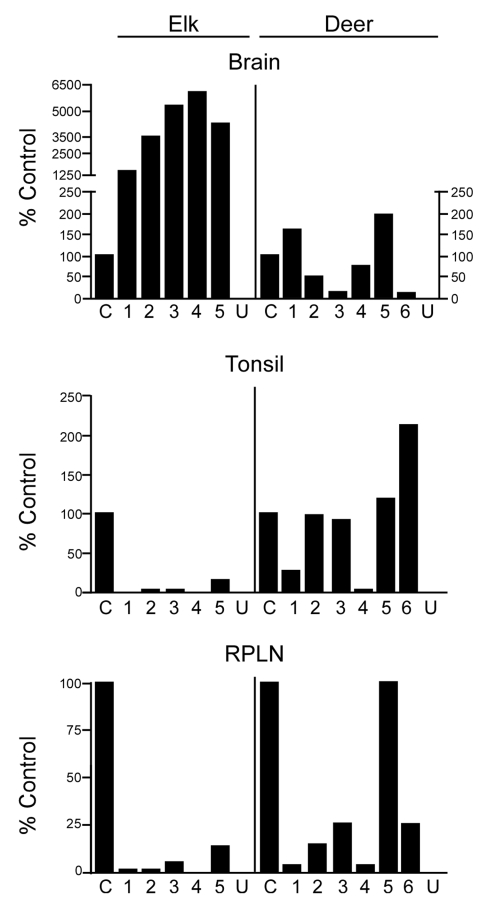
Quantification of disease-associated prion protein (PrPres) in brain, tonsil, and retropharyngeal lymph node (RPLN) from chronic wasting disease–affected elk and deer. The relative amount of PrPres in each lane of Figure 1 is shown relative to a common control described in the Figure 1 legend. A split scale is shown for elk brain because the PrPres signal from each elk brain was strong enough with 2-mg equivalents of tissue to obscure the protein patterns on the gel. Twenty-milligram equivalents were analyzed for all other tissues. The 10-fold difference in the amount of tissue equivalents loaded is accounted for by the split scale, where the result from 2-mg equivalents was multiplied by 10. Data shown are the average of 4 duplicate gels run for each sample. PrPres level in elk brain is significantly different from deer brain (p<0.001), elk tonsil is significantly different from deer tonsil (p = 0.0274), and elk RPLN is significantly different from deer RPLN (p = 0.0087) (Mann-Whitney test). C, reference control; U, uninfected elk or deer.

Tissues from 10 CWD-affected elk and 15 CWD-affected deer were analyzed in this study. All 10 of the elk had advanced clinical CWD when euthanized. The deer represented various stages of clinical disease. Detailed data showing PrPres glycoform profiles and quantification of PrPres are shown for 5 of the elk and 6 of the deer (Figure 1). Each of the elk brains gave a very strong PrPres signal when 2-mg brain equivalents were analyzed. Deer brain PrPres was more variable, and 20 mg of brain equivalent was analyzed (Figure 1). The relative amount of PrPres in each sample was determined by comparing the PrPres signal to a standard control. The standard control for each blot was RPLN from one of the CWD-infected mule deer in the study (labeled C in each blot, Figure 1). Relative PrPres amounts were determined by using a phosphor-imager and Image Quant software (Storm, Molecular Dynamics, Sunnyvale, CA, USA). The average amount of PrPres in elk brain was consistently higher than the amount in deer brain (Figure 2). The lower amounts of PrPres in deer brain than in elk brain likely reflects the more variable and earlier clinical status of the deer that were analyzed.

Diagnosis of CWD is often based on detection of PrPres in tonsil tissue by using ELISA and IHC analysis. Therefore, we also analyzed tonsil tissue, but because we were interested in quantitative issues we used immunoblot technology.

No PrPres was observed in tonsil from 2 of the elk (Figure 1B), and only a small amount was detected in tonsil from the other 3 elk (Figures 1B, 2B). PrPres in the 3 tonsil specimens that did give a signal averaged 4% of the control’s signal. In contrast, tonsil from 5 of the 6 CWD-affected deer gave a strong PrPres signal (Figure 1B), averaging 109% of the reference control’s signal. However, the tonsil of the remaining deer (#4, Figures 1B, 2B) gave no PrPres signal on immunoblot.

PrPres quantities in RPLNs from elk were also low. RPLN from 1 elk was negative (#4, Figure 1C), while weak reactions were seen for RPLNs from the other 4 elk at 2%, 2%, 5.1%, and 13% of the control, respectively (Figures 1C, 2C). RPLNs from the deer were much stronger. RPLNs from all 6 deer were positive and ranged from 3.4% to 100% of that of the control (Figures 1C, 2C). One of the deer (#4, Figure 1B, C) had no PrPres detected in tonsil and very little in RPLN, even though the reaction from the brain of this deer was strong. Tonsils and RPLNs from 5 additional CWD-infected elk and 9 additional CWD-infected deer, including 3 white-tailed deer, showed PrPres in amounts similar to those of most elk and deer shown in Figures 1 and 2, but detailed quantification was not carried out on these samples. The combined data for all elk and deer show tonsil and RPLN specimens to be consistently PrPres positive by immunoblot in deer but positive less frequently in elk (Table 1).

**Figure 3:**
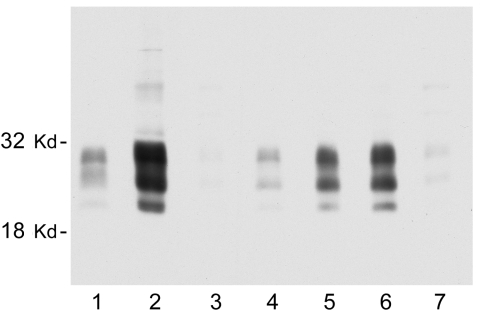
Representative immunoblot showing the relative amount of disease-associated prion protein (PrPres) in brain, tonsil, and selected lymph nodes from a single chronic wasting disease (CWD)–affected mule deer. All lanes were loaded with 10-mg equivalents of tissue (original wet weight basis). Lane 1, brain; lane 2, tonsil; lane 3, popliteal lymph node; lane 4, retropharyngeal lymph node (RPLN); lane 5, prescapular lymph node; lane 6, submandibular lymph node; lane 7, mesenteric lymph node. PrPres bands were visualized by using antibody L42 at 0.04 μg/mL and standard enhanced chemiluminescence processing.

We also sought to determine whether all of the nodes from individual deer contain similar levels of PrPres. Considerable variation was observed. In some deer, every node that was tested was PrPres positive, but more frequently only 1 or 2 nodes were positive. Furthermore, the intensity of the PrPres signals varied from node to node. In most deer, RPLN gave the strongest PrPres signal, but in other deer the prescapular or submandibular nodes were best (Figure 3). The mesenteric node was often positive, but generally gave a weak PrPres signal (Figure 3, lane 7). Thus, analysis of a single lymph node other than the RPLN by immunoblot would likely result in some CWD-positive deer being undetected.

The spleen has been shown to influence disease pathogenesis in both sheep and mouse models of TSE disease (*7*,*12*,*13*,*26*). Therefore, we also sought to quantify the amount of PrPres in elk and deer spleen. However, all of the animals gave very weak or no PrPres signals in spleen (Table 1) (blots not shown). Thus CWD-affected elk and deer differed from scrapie-affected sheep, in which the spleen routinely gives a strong PrPres signal.

### PrPres Glycoform Patterns in Lymphoid Tissues of CWD-infected Elk and Deer

**Figure 4:**
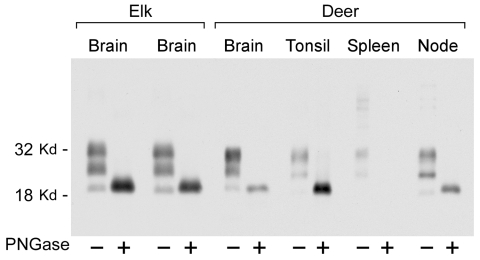
Immunoblot showing disease-associated prion protein from chronic wasting disease–affected elk brain or mule deer brain, tonsil, spleen, and retropharyngeal lymph node before and after treatment with PNGaseF. Alternating lanes show before and after treatment for each tissue. PNGaseF digestion was done as described in Materials and Methods. Ten-milligram equivalents of tissue were used for PNGase F–negative lanes, and 4-mg equivalents of tissue were used for PNGase F–positive lanes. The blot was developed as described in Figure 3.

PrPres glycoform patterns have been used to define TSE strains and have been studied extensively in deer and elk brain (*23*). Therefore, we evaluated the PrPres glycoform patterns of lymphoid tissues of CWD-infected deer and elk to identify profiles that might differentiate deer from elk. The glycoform profile in deer tonsil and lymph node were similar to that of the profile in deer brain (Figure 4). Likewise, there was no convincing difference in the pattern of PrPres found in deer and elk brains (Figure 1). A meaningful comparison of glycoform patterns between elk and deer tonsil and lymph nodes was not possible because none of the elk lymphoid organs gave a sufficiently strong PrPres signal.

Because PrPres band differences can be due both to differing glycosylation and different sites of proteinase cleavage, we treated samples of various tissues with PNGaseF to remove carbohydrates and thus show any differences due to proteolytic cleavages. Such differences in the PrPres structural core might provide evidence for the existence of different CWD strains as seen before in other TSE diseases (*27*–*29*). However, no differences were detected in PNGaseF-digested PrPres from elk and deer brain (Figure 4). Thus, both glycoform profiles and PNGaseF analysis indicated that PrPres from elk and deer were similar.

## Discussion

We found marked differences in the quantity of PrPres in tonsil and lymph nodes of CWD-infected elk versus deer by using immunoblot analysis. For example, PrPres was detected at high levels in deer, but not in elk, when 20-mg equivalents of tonsil or RPLN were analyzed. In contrast, brains from these deer and elk were all highly positive when the same immunoblot method was used. Both the quantitative PrPres difference (Figure 2) and the differences in the frequency of PrPres detection (Tables 1, 2) between elk brain and lymph nodes were not noted in previous studies in which nonquantitative ELISA or IHC methods were used (*5*,*18*,*19*). In these reports, most elk (85%–94%) had detectable PrPres in both brain and RPLN; however, 6%–15% of the elk had no PrPres in RPLN when brain was positive, which was similar to our immunoblot results. In fact, RPLN from most of the elk in our study were also positive by ELISA or IHC (data not shown). Thus, ELISA and IHC appeared to be more sensitive than immunoblot for PrPres detection and therefore more appropriate methods for diagnosis and surveillance. In contrast, immunoblotting appeared to be more useful for studies requiring quantitation or visualization of PrPres banding patterns.

Our results suggest that fundamental differences may exist in the pathogenesis of CWD between deer and elk. In CWD-infected deer, as with scrapie in sheep, infectivity and PrPres are detectable in lymphoid tissues early after infection, well before they can be detected in brain tissue (*7*,*16*). In deer and sheep, this early lymphoid involvement is considered important in the process of neuroinvasion and the kinetics of disease. After a period of replication in these peripheral sites, the infectious agent moves to the central nervous system. In elk, the low quantity of PrPres in tonsil or lymph nodes suggests that lymphoid infection may not necessarily precede neuroinvasion. Possibly the small amount of PrPres detected in elk tonsil and lymph node may actually originate from the brain. This situation may be similar to that of mink that have mink encephalopathy in which infection of peripheral lymphoid and other tissues is seen only when the animals are in the late stages of disease. It is unclear whether such spread from brain to the periphery is bloodborne or mediated by retrograde transmission through autonomic nerves (*31*).

In the TSE diseases in which lymphoid tissues are substantially involved, i.e., sheep scrapie and CWD in deer, horizontal transmission in natural situations is efficient. In contrast, when peripheral lymphoid tissues are not extensively involved, i.e., BSE in cattle (*14*,*15*), and naturally occurring CWD in elk, horizontal transmission appears to be relatively inefficient (Table 2). Thus, differences in lymph node PrPres levels correlate with differences in the prevalence of CWD in deer and elk in natural settings. This finding might be the result of greater quantities of CWD infectivity released to the environment from lymphoid tissues of deer that have died or been killed. Also because there is widespread distribution of large quantities of PrPres in deer lymphoid tissues, it seems possible that infectivity might also be present in other peripheral tissues such as intestine, kidney, or salivary glands, which could possibly lead to excretion or secretion of infectivity in feces, urine, or saliva. One would also expect brain-associated infectivity to be a source of environmental contamination, and in this regard brain from CWD-infected elk represents as great a risk as CWD-infected brain from deer.

**Table 2:** Comparison of PrPres tissue distribution in TSE-affected ruminants*†

Species (condition)	Brain	Spleen	Nodes	Tonsil	Natural transmission
Elk (CWD)	10/10	2/10	5/10	4/9	Low
Mule deer (CWD)	12/12	2/10	12/12	9/10	High
White-tailed deer (CWD)	3/3	1/3	3/3	2/3	High
Sheep‡ (scrapie)	8/8	7/8	6/8	Yes	High
Cattle (BSE)§	6/6	Neg	Neg	Neg	No

*PrPres, disease-associated prion protein; TSE, transmissible spongiform encephalopathy; CWD, chronic wasting disease; BSE, bovine spongiform encephalopathy; Neg, negative. †Distribution of PrPres or infectivity in peripheral tissues of TSE affected ruminants was compared. Data for deer and elk were determined in the study presented here. Data for sheep are from an earlier publication (*30*) as are the data for cattle (*14**,**15*) ‡Data from (*30*). §Data from (*14*).

Several other factors might also influence transmission within deer and elk populations. For example, differences in social interaction, the size of typical homeland range, preferred habitat, population densities, and so forth. The relative contribution of the possible factors is not known.

Although CWD prevalence in elk is low in natural settings, it can be much higher in confinement situations. What differences increase transmission when animals are confined is not known. Apparently, high PrPres levels in lymphoid tissues are not essential for transmission in crowded conditions. However, at least 2 factors might have an additional impact on transmission in captive elk. First, restricting elk to small pastures, sheds, or corrals where infectious material has accumulated over time might facilitate increased transmission. Second, in confined settings, animal-to-animal contact would increase. This might involve exchange of infectivity through saliva, which has been found to be infectious in deer (*32*) and might also be positive in elk, although this remains unproven.

Earlier studies have not shown any evidence for transmission of CWD to humans (*33*–*35*). CWD has been transmitted to cattle by intracerebral but not by oral inoculation (*36*), and no reports have found that co-pasturing of CWD-infected deer or elk with cattle has resulted in transmission. Furthermore, in vitro assays designed to test the susceptibility of humans or cattle to CWD suggested a very low probability of transmission to humans (*37*). Sheep, however, are likely to be more susceptible to CWD. They have been infected by intracerebral inoculation (*38*), and at a molecular level, CWD PrPres was shown to convert sheep PrP to the disease-associated form with relatively high efficiency (*37*). Thus, among livestock, sheep might be a possible target for CWD infection in appropriate situations such as co-pasturing. Also, a CWD agent from putatively infected sheep could have a host range not usually associated with CWD and might cross species barriers more readily than CWD from cervids. Thus, if CWD continues to expand in deer and elk populations, the possibility of transmission to noncervid species will require continued surveillance.
